# Diminished Frequencies of Cytotoxic Marker Expressing T- and NK Cells at the Site of *Mycobacterium tuberculosis* Infection

**DOI:** 10.3389/fimmu.2020.585293

**Published:** 2020-09-25

**Authors:** Gokul Raj Kathamuthu, Kadar Moideen, Rathinam Sridhar, Dhanaraj Baskaran, Subash Babu

**Affiliations:** ^1^International Center for Excellence in Research, National Institutes of Health, National Institute for Research in Tuberculosis, Chennai, India; ^2^National Institute for Research in Tuberculosis (NIRT), Chennai, India; ^3^Government Stanley Medical Hospital, Chennai, India; ^4^Laboratory of Parasitic Diseases, National Institute of Allergy and Infectious Diseases, National Institutes of Health, Bethesda, MD, United States

**Keywords:** tuberculous lymphadenitis, cytotoxic markers, lymph nodes, NK cells, CD4^+^ and CD8^+^ T cells

## Abstract

Tuberculous lymphadenitis (TBL) individuals exhibit reduced frequencies of CD8^+^ T cells expressing cytotoxic markers in peripheral blood. However, the frequencies of cytotoxic marker expressing CD4^+^, CD8^+^ T cells, and NK cells at the site of infection is not known. Therefore, we measured the baseline and mycobacterial antigen specific frequencies of cytotoxic markers expressing CD4^+^, CD8^+^ T cells, and NK cells in the LN (*n* = 18) and whole blood (*n* = 10) of TBL individuals. TBL LN is associated with lower frequencies of CD4^+^ T cells expressing cytotoxic markers (Granzyme B, CD107a) compared to peripheral blood at baseline and in response to PPD, ESAT-6, and CFP-10 antigen stimulation. Similarly, lower frequencies of CD8^+^ T cells expressing cytotoxic markers (Perforin, Granzyme B, and CD107a) were also present in the TBL LN at baseline and following (except perforin) antigen stimulation. Finally, at baseline and after antigen (PPD, ESAT-6, and CFP-10) stimulation, frequencies of NK cells expressing cytotoxic markers were also significantly lower in TBL LN compared to whole blood. Hence, TBL is characterized by diminished frequencies of cytotoxic marker expressing CD4^+^, CD8^+^ T cells, and NK cells at the site of infection, which might reflect the lack of protective immune responses at the site of *Mycobacterium tuberculosis* infection.

## Introduction

Cytotoxic T lymphocytes (CTL) are mainly involved in the killing of macrophages (Mφ) infected cells with *Mycobacterium tuberculosis* (Mtb) (1, 2). Natural killer (NK) cells are the crucial element of innate immunity against Mtb and mediate cytotoxicity and cytokine signaling as well as form a bridge between innate and adaptive immune responses (3, 4). Different NK cell subsets have been described and have the ability to induce cell-mediated cytotoxicity and cytokine production at various levels (5). They also mediate the elimination of infected cells through antibody-dependent cellular cytotoxicity (ADCC) and non-MHC-restricted cytotoxic mechanism (6–8). In addition, CD4^+^ T helper (Th) cells are equally important for the host protection against Mtb by expressing interferon γ (IFNγ). Therefore, deficiencies in the production of both IFNγ and interleukin (IL)-12 highly elevate the risk of tuberculosis (TB) disease. They are also important for stimulating the antimicrobial action of macrophages (9, 10). Like CD4, CD8^+^ T cells are also significantly important in providing resistance to Mtb infection by activating various [IFNγ, tumor necrosis factor (TNFα), IL-2] cytokines and by eliminating Mtb infected macrophages (11–13). They also have the ability to induce cytolytic activity through granule (i.e., perforin, granzyme, and granulysin) production (14, 15).

Both mice and human data revealed that cytotoxic effector molecules have the ability to lyse the target cells either by ligand-ligand cell death pathway or by the delivery of cytolytic molecules translocated directly in to the cytoplasmic granules (16–19). In addition, lipid bilayer containing lysosomal-associated membrane glycoproteins (LAMP1 or CD107a) is a T cell degranulation marker which helps in the elimination of Mtb infected cells (20, 21). Even at the infection site, Mtb interacts with various antigen-presenting cells in order to mediate anti-Mtb immunity and coordinates the presentation of TB antigens to CD4^+^ and CD8^+^ T cell in the lymph nodes (22).

TBL is the most frequent form of extra-pulmonary TB (EPTB) with the prevalence rate of 15 to 20% among all TB cases in India and more common in women than men (23, 24). Previous data revealed that TBL disease associated with higher frequencies of baseline and antigen stimulated CD8^+^ T cells expressing Type 1 (IL-2, TNFα) and Type 17 (IL-17A, IL-17F) cytokines than PTB (25). We have also recently shown that TBL individuals are associated with reduced antigen specific expression of TNFα and enhanced IL-17F levels at the site of infection compared to peripheral blood (26). However, until now, no human research studies have examined the frequencies of T cells and NK cells expressing cytotoxic (perforin, granzyme B, and CD107a) markers in tuberculous lymphadenitis (TBL), specifically at the site of infection i.e., in the affected lymph nodes. Thus, in the present study, we have examined the frequencies of CD4^+^ and CD8^+^ T cells and NK cells expressing cytotoxic markers in the lymph nodes and compared them with peripheral blood of TBL individuals. We report that TBL LN is associated with significantly diminished frequencies of CD4^+^, CD8^+^ T cells and NK cells expressing cytotoxic markers than the peripheral blood.

## Results

### TBL Is Associated With Diminished Baseline and Antigen-Specific Frequencies of CD4^+^ T Cells Expressing Cytotoxic Markers in LN

**FIGURE 1:**
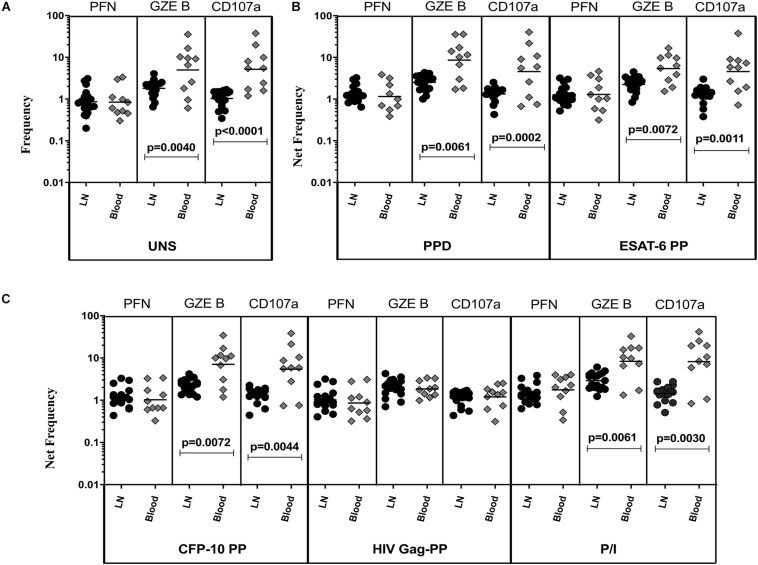
Diminished baseline and antigen-specific frequencies of CD4^+^ T cells expressing GZE B and CD107a in TBL lymph nodes. Lymph node (*n* = 18) and whole blood (*n* = 10) from TBL individuals were cultured with medium alone or mycobacterial or control antigens for 18 h. **(A)** Baseline, **(B)** PPD, ESAT-6 PP, and **(C)** CFP-10 PP, HIV Gag PP, and P/I antigen stimulated frequencies of CD4^+^ T cells expressing respective cytotoxic (PFN, GZE B, and CD107a) markers in lymph node (each dark round circle represents single individuals) versus blood (each gray diamond boxes circle represents single individuals). The bar represents the geometric mean values and *P-*values were calculated using the Mann-Whitney *U* test. The net frequencies were calculated by subtracting the baseline from antigen-stimulated values for each individual.

To determine the cytotoxic marker expressing CD4^+^ T cells in TBL individuals, we used multi-color flow cytometry to define the frequencies of baseline and mycobacterial antigen-specific stimulated CD4^+^ T cells expressing perforin (PFN), granzyme B (GZE B), and CD107a (Figure 1). The gating strategy of CD4^+^, CD8^+^ T cells, and NK cell is shown in Supplementary Figure S1. Similarly, the representative plot of CD4^+^ T cells expressing peripheral blood (A) and lymph node (B) cytotoxic markers is given in Supplementary Figure S2. As shown in Figure 1A, in unstimulated (UNS) condition, the frequencies of CD4^+^ T cells expressing GZE B and CD107a were significantly diminished in the LN compare to whole blood of TBL individuals. Similarly, net frequencies of CD4^+^ T cells expressing GZE B were significantly decreased upon PPD (Figure 1B), ESAT-6 PP (Figure 1B) and CFP-10 PP (Figure 1C) antigen stimulation in TBL LN when compared to whole blood of TBL individuals. In contrast, the net frequencies of CD4^+^ T cells expressing PFN was not significantly different between LN and whole blood in UNS and also in other antigen stimulated condition (Figures 1A–C). Similarly, in response to HIV Gag PP antigen, there were no significant differences in the net frequencies of cytotoxic markers (Figure 1C). Finally, upon P/I stimulation, frequencies of CD4^+^ T cells expressing GZE B and CD107a were significantly diminished in LN when compared to whole blood of TBL individuals (Figure 1C). Therefore, LN of TBL individuals is associated with diminished frequencies of CD4^+^ T cells expressing cytotoxic (GZE B, CD107a) markers.

### TBL Is Associated With Diminished Baseline and Antigen-Specific Frequencies of CD8^+^ T Cells Expressing Cytotoxic Markers in LN

**FIGURE 2:**
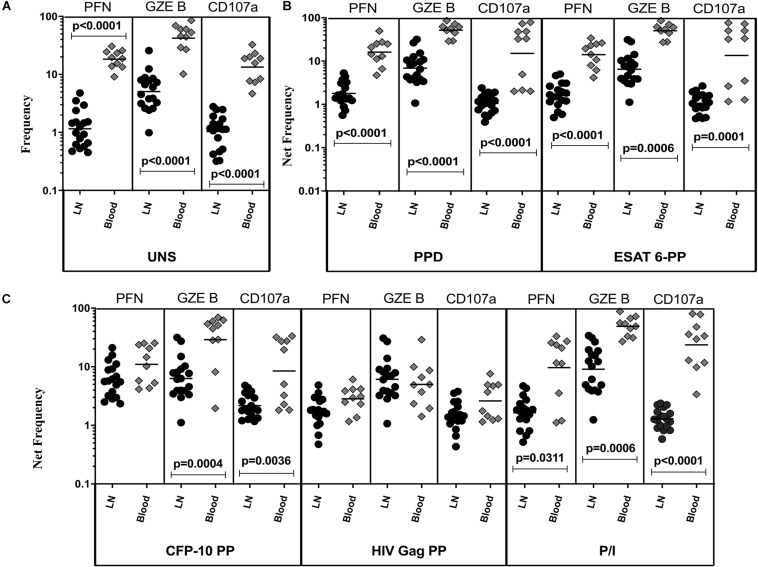
Diminished baseline and antigen-specific frequencies of CD8^+^ T cells expressing cytotoxic markers in TBL lymph nodes. Lymph node (*n* = 18) and whole blood (*n* = 10) from TBL individuals were cultured with medium alone or mycobacterial or control antigens for 18 h. **(A)** Baseline, **(B)** PPD, ESAT-6 PP, and **(C)** CFP-10 PP, HIV Gag PP, and P/I antigen stimulated frequencies of CD8^+^ T cells expressing respective cytotoxic (PFN, GZE B, and CD107a) markers in lymph node (each dark round circle represents single individuals) versus blood (each gray diamond boxes circle represents single individuals). The bar represents the geometric mean values and *P-*values were calculated using the Mann-Whitney *U* test. The net frequencies were calculated by subtracting the baseline from antigen-stimulated values for each individual.

The representative plot of CD8^+^ T cells expressing peripheral blood (A) and lymph node (B) cytotoxic markers is given in Supplementary Figure S3. To define the cytotoxic marker expressing CD8^+^ T cells in TBL disease, we measured the frequencies of baseline and mycobacterial antigen-specific CD8^+^ T cells expressing PFN, GZE B, and CD107a (Figure 2). We observed significantly decreased frequencies of CD8^+^ T cells expressing PFN, GZE B, and CD107a at baseline of TBL LN compared with TBL whole blood (Figure 2A). Similarly, in response to PPD (Figure 2B), ESAT-6 PP (Figure 2B) and CFP-10 PP (Figure 2C), there were significantly reduced net frequencies of CD8^+^ T cells expressing PFN (except CFP-10 PP), GZE B and CD107a in LN compared with peripheral blood of TBL individuals. Like CD4^+^ T cells, net frequencies of CD8^+^ T cells expressing cytotoxic markers were not significantly different between LN and whole blood of TBL individuals after stimulation with HIV Gag PP antigen (Figure 2C). Finally, upon stimulation with P/I, there were reduced net frequencies of CD8^+^ T cells expressing PFN, GZE B and CD107a in LN of TBL individuals (Figure 2C). Therefore, LN of TBL individuals is associated with diminished frequencies of CD8^+^ T cells expressing cytotoxic markers.

### TBL Is Associated With Diminished Baseline and Antigen-Specific Frequencies of NK Cells Cell Expressing Cytotoxic Markers in LN

**FIGURE 3:**
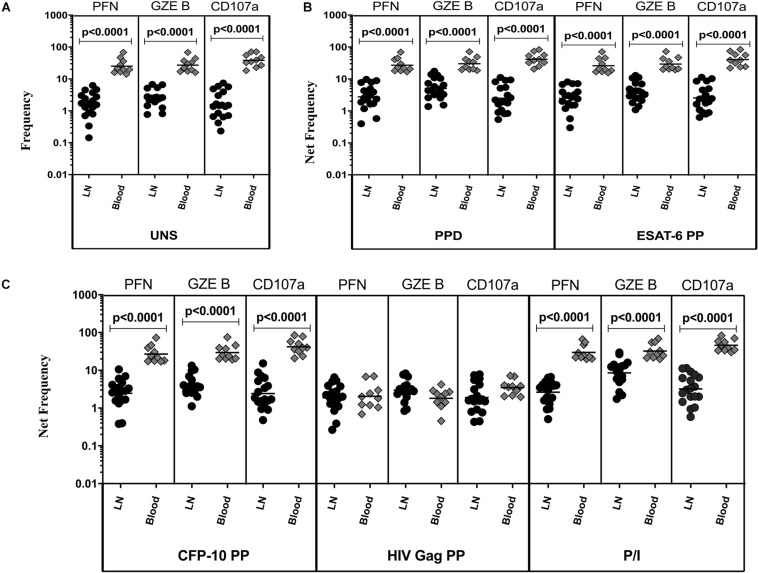
Diminished baseline and antigen-specific frequencies of NK cells expressing cytotoxic markers in TBL lymph nodes. Lymph node (*n* = 18) and whole blood (*n* = 10) from TBL individuals were cultured with medium alone or mycobacterial or control antigens for 18 h. **(A)** Baseline, **(B)** PPD, ESAT-6 PP, and **(C)** CFP-10 PP, HIV Gag PP, and P/I antigen stimulated frequencies of NK cells expressing respective cytotoxic (PFN, GZE B, and CD107a) markers in lymph node (each dark round circle represents single individuals) versus blood (each gray diamond boxes circle represents single individuals). The bar represents the geometric mean values and *P-*values were calculated using the Mann-Whitney *U* test. The net frequencies were calculated by subtracting the baseline from antigen-stimulated values for each individual.

The representative plot of NK cells expressing peripheral blood (A) and lymph node (B) cytotoxic markers is given in Supplementary Figure S4. To determine cytotoxic marker expressing NK cells in TBL disease, we have assessed the frequencies of baseline and mycobacterial antigen-specific NK cells expressing PFN, GZE B, and CD107a (Figure 3). As shown in Figure 3A, in UNS condition, the frequencies of NK cells expressing cytotoxic (PFN, GZE B, and CD107a) markers were significantly reduced in the LN of TBL when compared to the peripheral blood. Similarly, after stimulation with PPD (Figure 3B), ESAT-6 PP (Figure 3B) and CFP-10 PP (Figure 3C), net frequencies of NK cells expressing cytotoxic (PFN, GZE B, and CD107a) markers were significantly reduced in LN of TBL compare to whole blood. As shown in Figure 3C, upon stimulation with HIV Gag PP, the net frequencies of NK cells expressing cytotoxic markers were not significantly different between LN and whole blood of TBL individuals. Finally, upon stimulation with P/I, there were reduced net frequencies of NK cells expressing cytotoxic (PFN, GZE B) markers in TBL LN (Figure 3C). Therefore, LN of TBL individuals is associated with diminished frequencies of NK cells expressing cytotoxic (PFN, GZE B, and CD107a) markers.

## Discussion

The importance of cytotoxic markers in the pathogenesis of EPTB disease is less studied especially at the site of infection i.e., in the affected lymph nodes (LN). Hence in this present study, we have examined the frequencies of CD4^+^, CD8^+^ T cell, and NK cell cytotoxic markers in both the peripheral blood and LN of TBL individuals. We demonstrate that LN of TBL is associated with significantly diminished frequencies of CD4^+^, CD8^+^ T cell, and NK cell expressing cytotoxic markers when compared to whole blood. Different cytotoxic mechanisms such as interaction of Fas/FasL, lysis/apoptosis of infected cells by cytotoxic granule toxins are crucial in governing TB infection (27). Similarly, CD8^+^ T cells are very crucial to control Mtb infection and also co-ordinate various other functions to boost the host immune responses (11). NK cells also possess cell-mediated cytotoxicity and aid in forming bridge between the innate and adaptive immune system (3, 4, 6, 28).

Our data demonstrates that PFN expressing CD4^+^ T cell frequencies did not differ significantly between whole blood and lymph nodes of TBL. This result was consistent with un-stimulated and even in different TB antigen (PPD, ESAT-6 PP, and CFP-10 PP) stimulated conditions as well. In contrast, CD4^+^ T cell frequencies of GZE B and CD107a were significantly diminished in LNs of TBL individuals upon no stimulation and antigen specific (PPD, ESAT-6 PP, and CFP-10 PP) stimulation. Deficiency of perforin *in vivo* causes reduced cytotoxicity ability against TB and perforin is a key molecule in the control of TB disease (16). Similarly, GZE B can induce cytotoxicity without PFN but their frequencies were reduced in LN suggesting they were deficient in their ability to fight against TB infection. Different studies have shown reduced or elevated frequencies of CD8^+^ T cell expressing PFN levels in active TB compared to healthy controls (29, 30). Conversely, another study has shown the diminished frequencies of CD8^+^ T cell expressing PFN and GZE B in whole blood of TBL individuals than active TB (25). Our findings also disclosed a similar result by showing the significantly downregulated frequencies of PFN and GZE B but not CD107a in LN of TBL compared with periphery in UNS and also with all TB (PPD, ESAT 6-PP, and CFP-10 PP) antigen and P/I antigen stimulated conditions. Apart from perforin, both mice and human data suggest that CD8^+^ T cell mediated cytotoxicity needs GZE B molecule for the stimulation of cell death occurs via granule exocytosis pathway. GZE B is very important in the activation of cytokines specifically the IL-1 family cytokines (31–33). Similarly, T regulatory cells (Tregs) obtained from GZE B knockout mice co-cultured with effector T cells have less efficient induction of the former than the wild type mice (34). Our data corroborates the same where decreased frequencies of PFN and GZE B might fail to induce their cytotoxic ability and their immunological deficit might result in the persistence of higher infection rate in LNs of TBL patients.

Finally, we revealed that the frequencies of NK cell expressing PFN, GZE B, and CD107a cytotoxic markers are significantly lower in LN compared to whole blood with UNS and after stimulation with TB antigens (PPD, ESAT-6 PP, and CFP-10 PP) and P/I antigen, suggesting the inability to exert their cytotoxic potential against TB infection. NK cells express both perforin and granulysin cytotoxic markers to kill Mtb through the direct interaction with the mycobacteria (28). GZE B also regulates the IL-1α cytokine extracellularly in order to reduce the non-specific leakage during the NK cell mediated killing (35). This observation indicates the ongoing immune alterations in the host of infected individuals especially at the site of infection. GZE B could potentially induce receptor-mediated endocytosis which facilitates their entry into the cytoplasm of the specific target cell to encourage apoptosis mediated cell death (36, 37). To support our findings, similar results were found in active PTB patients and shown decreased frequencies of GZE B after stimulation with ESAT-6 and CFP-10 peptides (29). Further it is also supported by other studies in active TB and healthy individuals by showing the frequencies of effector molecules were greatly reduced (6, 38). Likewise, deficient NK cells expressing GZE B exhibit a severe flaw activation of apoptosis in infected cells and unexpected defect in ^51^Cr release (37). CD107a have an important role in trafficking of perforin inside the vesicles and their deficiency inhibits the transport of perforin in to the lytic granules (39, 40). Hence, the loss of cytotoxic markers expression in the LNs might impair their effective activation against Mtb infection. The other potential reason might be TBL is a paucibacillary form of TB disease where higher antigen load is present in the site of infection and thus higher bacterial infection reduces the potential action of cytotoxic markers.

The cytotoxic markers expression (PFN, GZE B, and CD107a) is an indirect measure to analyze the cytotoxic potential of CD4^+^, CD8^+^ T cells and NK cell (41). Intriguingly, the reduced frequencies were also observed with P/I stimulation indicating a cell intrinsic effect of cytotoxic markers. Our data reveals that cytotoxic marker expression is impaired at the site of infection (in the affected LN) of TBL individuals and Mtb might utilize this mechanism to escape from the host immune mediated protection. Our study has certain limitations specifically, the study groups were small and thus repeating the examination with larger study cohorts would be helpful to understand their function. The other limitations of our study are not including the control (non-lymphadenitis) group or carry out the *in vitro* functional experiments using TBL samples. Overall, the down regulation of cytotoxic markers at the site of infection is a hallmark of this poorly documented form of TB.

## Materials and Methods

### Ethics Statement

The present study was sanctioned by Internal Ethics Committees of National Institute of Research in Tuberculosis (NIRT, NIRTIEC2010007) and informed written consent was obtained from all participants.

### Study Patients

We studied a group of 18 individuals with TBL disease. TBL diagnosis was made on the basis of culture positivity for *Mycobacterium tuberculosis* from the excised lymph node biopsy samples. All the study patients were HIV negative and devoid of any steroid treatment. The baseline blood samples were collected and then they were treated with standard anti-tuberculosis regimen for 6 months. Blood and lymph nodes were isolated from the same set of individuals. The patient demographics were included in Table 1.

**TABLE 1:** Demographics of the study population.

Study demographics	TBL blood	TBL Lymph node
No of subjects recruited (n)	10	18
Gender (M/F)	4/6	7/11
Median age in years (range)	27.8 (18–47)	28.4 (18–51)
Lymph node culture grade (0/1+/2+/3+)	2/8/0/0	2/14/2/0
Smear Grade (1+/2+/3+)	Not done	Not done

### Antigens

Purified protein derivative (PPD; Statens Serum Institute, 10 μg/mL), recombinant early-secreted antigen 6 (ESAT-6, 10 μg/mL) and culture filtrate protein 10 (CFP-10, 10 μg/mL) peptide pools [BEI Resources, National Institute of Allergy and Infectious Diseases (NIAID), National Institutes of Health (NIH)] were the antigens used in the study. The human immunodeficiency virus Gag peptide pool (HIVPP; AIDS Reagent Program, Division of AIDS, NIAID, NIH, 10 μg/mL) was used as non-TB specific antigen stimuli i.e., negative control. Finally, the combination of Phorbol 12-myristate 13-acetate (P)–ionomycin (P/I; Calbiochem, 12.5 ng/mL and 125 ng/mL) was used as a positive control.

### Whole Blood and Lymph Node Culture

The *in vitro* cell cultures were performed to measure cytotoxic marker levels. Briefly, the whole blood was diluted as 1:1 with the Roswell Park Memorial Institute medium (RPMI) 1640 medium provided with penicillin-streptomycin (100 U/100 mg/mL), L-glutamine (2 mM), and HEPES (10 mM) (Invitrogen, San Diego, CA, United States) buffer. The excised lymph nodes from the patients were harvested in RPMI 1640 medium and processed immediately. The samples were washed twice and cut down into smaller pieces in RPMI 1640 medium were further treated with Liberase (0.1 mg/mL) and DNase (0.1 mg/mL) enzymes (Roche Diagnostics). Then the LN was incubated for 20 to 30 min at 37°C. Again, the cells were washed with RPMI 1640 medium and centrifuged at 2,600 rpm for 10 min and the supernatant was discarded. The samples were uniformly dispersed (2 mL/well) in 12-well tissue culture plates (Costar; Corning Inc., Corning, NY, United States). Whole blood and lymph node cells were stimulated with antigens such as PPD, ESAT-6, CFP-10, HIVPP, P/I or were left unstimulated (UNS) along with CD49d/CD28 co-stimulatory (only for whole blood) and incubated at 37°C in 5% CO_2_ for 18 h. After 2 h of incubation, Fast Immune^TM^ brefeldin A solution (10 μg/mL) was added to the cultures. After 18 h of incubation, the stimulated cells were meticulously transferred to 50 mL sterile falcon tubes. The samples were centrifuged at 2,600 rpm for 10 min. Further the samples were washed with 1x Phosphate-buffered saline (PBS-LONZA) and red blood cells were lysed using BD FACS^TM^ lysing solution. Finally, the cells (whole blood and lymph node) were fixed using BD cyto fix/cyto perm^TM^ and cryopreserved at −80°C in PBS/Dimethyl sulfoxide [DMSO (HiMedia)].

### Intracellular Cytokine Staining and Flow Cytometry

The cells were thawed, washed with 1x PBS, permeabilized using permeabilization buffer^TM^ (eBioscience) and incubated for 60 min. The cells were washed and permeabilization buffer^TM^ were added, stained with surface and cytotoxic markers and incubated overnight at 4°C. Cells were stained with surface markers like CD3 (BD Biosciences), CD4 (BD Biosciences), CD8, (BD Biosciences) and CD56 (eBioscience) and intracellular cytotoxic markers such as perforin (BD Pharmingen), granzyme B (Invitrogen-eBioscience) and CD107a (BD Biosciences) were used and incubated. After incubation, the cells were washed with ebioscience buffer and finally the cells were added with PBS. The acquisition was done using eight-color flow cytometry on a FACSCanto II flow cytometer with FACSDiva software v.6 (Becton Dickinson and Company, Cockeysville, MD, United States). We used the forward vs. side scatter to set the lymphocyte gating and 100,000 gated lymphocyte events were acquired. The data were gathered and analyzed with the help of Flow Jo software (Tree Star Inc., Ashland, OR, United States). The gating strategy for CD4^+^, CD8^+^ T, and CD56^+^ expressing cytotoxic markers was determined by FMO and the data were represented as frequencies (unstimulated) and net frequencies (antigen stimulations).

### Data Analysis

All the statistical analyses were accomplished using GraphPad PRISM (version 8) software (GraphPad Software, Inc., San Diego, CA, United States). The geometric means (GM) were used to assess the central tendency and intergroup comparisons were measured by non-parametric Mann-Whitney *U* test.

## Data Availability Statement

All datasets presented in this study are included in the article/Supplementary Material.

## Ethics Statement

The studies involving human participants were reviewed and approved by the Internal Ethics Committees of National Institute of Research in Tuberculosis (NIRT, NIRTIEC2010007) and informed written consent was obtained from all participants.

## Author Contributions

GK and SB conceived and designed the experiments and wrote the manuscript. GK and KM performed the experiments. GK analyzed the data. DB, RS, and SB contributed materials, reagents, and analysis tools. All authors contributed to the article and approved the submitted version.

## Conflict of Interest

The authors declare that the research was conducted in the absence of any commercial or financial relationships that could be construed as a potential conflict of interest.

## Supplementary Material

FIGURE S1 Gating strategy for TBL individuals **(A)** CD4^+^, CD8^+^, and CD56^+^ cell population gated on peripheral blood **(B)** CD4^+^, CD8^+^, and CD56^+^ cell population gated on lymph nodes.

FIGURE S2 Representative plot of CD4^+^ T cells expressing cytotoxic markers in peripheral blood and lymph nodes of TBL individuals. The intracellular assay of flow data showing the frequencies of cytotoxic (perforin, GZE B, and CD107a) markers **(A)** peripheral blood and **(B)** lymph nodes. The plots shown here are gated on CD3^+^CD4^+^ T cells.

FIGURE S3 Representative plot of CD8^+^ T cells expressing cytotoxic markers in peripheral blood and lymph nodes of TBL individuals. The intracellular assay of flow data showing the frequencies of cytotoxic (perforin, GZE B, and CD107a) markers **(A)** peripheral blood and **(B)** lymph nodes. The plots shown here are gated on CD3^+^CD8^+^ T cells.

FIGURE S4 Representative plot of NK cell expressing cytotoxic markers in peripheral blood and lymph nodes of TBL individuals. The intracellular assay of flow data showing the frequencies of cytotoxic (perforin, GZE B, and CD107a) markers **(A)** peripheral blood and **(B)** lymph nodes. The plots were shown here are gated on CD3^–^CD56^+^ T cells.
